# Combined Biofortification of Durum Wheat with Zinc and Selenium: Effects on Semolina and Pasta Nutrient Accumulation and Yield

**DOI:** 10.1007/s12011-026-05060-2

**Published:** 2026-03-24

**Authors:** Carlos García-Latorre, Angélica Rivera-Martín, María Dolores Reynolds-Marzal, Maria J. Poblaciones

**Affiliations:** Departamento de Ingeniería Medio Agronómico y Forestal, Escuela de Ingenierías Agrarias, Universidad de Extremadura, Avenida Adolfo Suárez s/n, 06007 Badajoz, Spain

**Keywords:** Fertilization, Mineral retention, Phytic acid, Zinc absorption modelling, Processing losses

## Abstract

Agronomic biofortification of durum wheat with selenium (Se) and zinc (Zn) represents a promising strategy to combat micronutrient deficiencies in human populations relying on cereal-based diets. This study evaluated the effectiveness of soil Zn application (S0Zn: no application; S50Zn: 50 kg of ZnSO_4_·7H_2_O ha^− 1^) and foliar applications of Se and Zn (0 F: no application; ZnF: two applications of 4 kg ZnSO_4_·7H_2_O ha^− 1^; SeF: 10 g Na_2_SeO_4_ ha^− 1^; ZnF + SeF: combined ZnF and SeF) on nutrient accumulation and yield in durum wheat semolina and pasta under Mediterranean rainfed conditions over two growing seasons. Semolina and pasta were analyzed for nutrient concentrations, phytic acid content, and indices of Zn bioavailability, including phytic acid–Zn molar ratios and modelled fractional and total absorbed Zn (FAZ and TAZ). Foliar treatments significantly increased Zn and Se concentrations in both semolina and pasta, with the combined Zn + Se treatment reaching 36.7 mg Zn kg⁻¹ and 43.1 µg Se kg⁻¹ in semolina and 32.7 mg Zn kg⁻¹ and 41.6 µg Se kg⁻¹ in pasta. These treatments also reduced PA: Zn ratios and increased estimated Zn absorption, while improving semolina yield by up to 4%. Processing from grain to semolina and pasta resulted in moderate but consistent micronutrient losses, with retention rates ranging from 66 to 74%. Overall, combined foliar application of Zn and Se proved to be an effective strategy to improve the nutritional quality of durum wheat-derived products under Mediterranean conditions.

**Supplementary Information:**

The online version contains supplementary material available at 10.1007/s12011-026-05060-2.

## Introduction

Durum wheat (*Triticum turgidum* ssp. *durum*) is an important staple crop, particularly in the Mediterranean region, which accounts for 75% of its global production [1, 2]. Beyond its role as a primary source of dietary energy, durum wheat is valued for its relatively high protein content (12–15%) and unique gluten properties, which make it particularly suitable for pasta production [3, 4]. In addition, durum wheat-based products contribute to the intake of essential minerals, vitamins, dietary fiber, and bioactive compounds, thereby playing a relevant role in human nutrition [5–7].

Despite its agronomic and nutritional importance, durum wheat, like many cereal crops, is often characterized by low concentrations of essential micronutrients, particularly selenium (Se) and zinc (Zn) [8–11]. This limitation is closely linked to the low availability of these elements in many agricultural soils. Approximately one-third of arable soils lack adequate levels of bioavailable Zn and Se [12]. Their bioavailability in soil is influenced by factors such as parent material composition, organic carbon concentration, soil pH, and anthropogenic activities [13]. In regions such as the southwestern Iberian Peninsula, considerably low levels of Se (~ 140 µg kg^− 1^) and Zn (< 25 mg kg^− 1^) have been observed [14–16], levels that are considered deficient according to established agronomic thresholds [17, 18].

As a result, crops cultivated on these soils frequently accumulate insufficient amounts of Se and Zn in edible tissues, leading to a reduced transfer of these micronutrients along the soil–plant–food chain [19]. This has important implications for human nutrition, as Se and Zn deficiencies are estimated to affect approximately two billion and one billion people worldwide, respectively [11, 20]. Selenium is critical for antioxidant defense, thyroid hormone metabolism, and immune function [21, 22], while Zn plays key roles in over 300 enzymes and 1000 transcription factors [23]. Deficiencies in these micronutrients are associated with severe health issues such as cardiovascular disease, impaired cognitive function, and compromised immunity [20, 22, 24, 25].

Biofortification has therefore emerged as a promising strategy to address micronutrient deficiencies by increasing the concentration of essential elements in edible plant tissues [26–28]. Among the different approaches, agronomic biofortification through soil and foliar fertilization offers a flexible and readily applicable option that can be rapidly implemented in existing cropping systems, including durum wheat [29–31].

Recent studies have suggested that the combined application of Se and Zn may provide additional benefits, improving micronutrient accumulation while maintaining yield and technological quality [26]. However, the effectiveness of combined Se and Zn biofortification is strongly influenced by environmental conditions, soil properties, and crop management practices. Moreover, in durum wheat, most studies have focused primarily on grain micronutrient concentrations, while considerably less attention has been paid to the fate of these elements during milling and processing into semolina and pasta, which ultimately determine the nutritional value of the consumed products. The milling process that transforms durum wheat into semolina, a key factor in the overall quality of the final pasta product, can lead to significant losses of minerals and vitamins naturally present in the whole grain. In this sense, reported losses during the conversion from whole grain to semolina range from 40 to 80% for iron (Fe), Zn, copper (Cu), and magnesium (Mg) [30, 32]. Furthermore, the nutritional effectiveness of these micronutrients in the final product depends not only on their concentration but also on their potential absorption, which can be limited by antinutritional factors such as phytic acid [33, 34]. Although combined Zn and Se biofortification has been previously explored in cereals, including under Mediterranean conditions, there remains a lack of integrated studies that evaluate micronutrient enrichment across the grain–semolina–pasta chain while accounting for interannual climatic variability and potential implications for proxy indicators of Zn bioavailability in final consumer products. Addressing these gaps is particularly relevant under Mediterranean rainfed conditions, where yield stability and nutrient accumulation are strongly affected by rainfall and temperature fluctuations.

Therefore, the objective of this study was to evaluate the effect of soil and foliar applications of zinc and selenium on micronutrient accumulation, indices of zinc absorption, and yield of durum wheat semolina and pasta grown under Mediterranean rainfed conditions.

## Materials and Methods

### Field Experiment and Harvest

The durum wheat used in this study was obtained from a field experiment conducted over two consecutive growing seasons (2017/18 and 2018/19) in southern Spain (38° 32’ 47.27’’ N, 6° 58’ 10.47’’ W; 186 m above sea level), under rainfed Mediterranean conditions. The exact geographical location of the experimental area is shown in Figure S1. The two growing seasons exhibited significant climatic variability, with 2017/18 receiving near-average rainfall (477 mm) including unusually high precipitation in March and April, while 2018/19 was considerably drier with only 295 mm total rainfall and pronounced drought periods, particularly in late winter and spring. Climatic conditions during the two growing seasons were monitored using a nearby meteorological station (Figure S2).

Prior to the establishment of the experiment, soil samples were collected from the 0–30 cm soil layer across the experimental field. Samples were air-dried, sieved (< 2 mm), and analyzed to determine their main physicochemical properties. Soil texture was determined by the gravimetric method, and pH was measured in a soil–water suspension (1:2.5, w/v) with a calibrated pH meter. Organic matter content (%) was determined by the potassium dichromate oxidation method [35], while total N (%), extractable P, and K, determined by Kjeldahl [36], Olsen [37], and ammonium acetate (1 N) [38] methods, respectively. For micronutrient analysis, DTPA extraction followed by inductively coupled plasma mass spectrometry (ICP-MS; Agilent 7500ce) was employed. The results are presented in Table 1.

**Table 1:** Physicochemical properties and baseline micronutrient concentrations of the soil (0–30 cm depth) at the experimental site before establishment of the field experiment

Soil parameter	Value
Texture	Clay loam
pH	6.4 ± 0.2
Organic matter content (%)	1.31 ± 0.09
Total N	0.120 ± 0.007
Extractable P (g kg^− 1^)	4.90 ± 0.05
Extractable N (mg kg^− 1^)	321 ± 8
Se content (mg kg^− 1^)	1.27 ± 0.01
Zn content (mg kg^− 1^)	0.303 ± 0.030

The experimental design was a split-plot with four randomized replications and a plot size of 15 m^2^ (3 × 5 m), with 0.5 m alleys to separate the plots. To avoid border effects, an inner area of 2 × 4 m was used for plant sampling and harvest. The main plot factor was soil Zn application (S0Zn: no application; S50Zn: 50 kg of ZnSO_4_·7H_2_O ha^− 1^ applied before the sowing), and the subplot factor was foliar application (0 F: no application; ZnF: two foliar applications of 4 kg ZnSO_4_·7H_2_O ha^− 1^ each at a two-week interval during stem elongation; SeF: foliar application of 10 g Na_2_SeO_4_ ha^− 1^ at booting stage; ZnF + SeF: a combination of ZnF and SeF treatments). The soil Zn application was performed only at the beginning of the first study year. Grain was harvested in early July of both years and properly preserved until processing.

### Sample Processing. Semolina Production and Pasta Making

Semolina yield was determined through a two-stage conditioning process. To determine the semolina yield, the durum wheat samples underwent conditioning and milling processes. Initially, the moisture content was measured using an Aquasearch PM 600 moisture tester (Kett, Florida, US). Subsequently, 500 g of the sample was placed in a plastic container, and water was added to achieve a moisture content of 16.5%. To ensure uniform moisture distribution throughout the sample, a Chopin MR2L Rotary Mixer (CHOPIN Technologies, Villeneuve-la-Garenne, France) was employed for 30 min, after which the sample was left to rest for three hours. Following the same procedure, the samples were then conditioned to 17.5% moisture content and allowed to rest for 24 h. After completing the sample preparation, milling was performed using a Chopin CD2 mill (CHOPIN Technologies, Villeneuve-la-Garenne, France), followed by sieving with a Sassor (Group Tripette & Renaud, France) to obtain semolina with particle sizes ranging from 150 to 500 μm. Semolina yield was calculated using the formula:

$$Yield\left(\%\right)=Ms*\frac{100-Hs}{Mt*\left(100-Ht\right)}*100,$$

where Ms and Mt are masses of semolina and grain, and Hs and Ht are their respective moisture contents.

Pasta production was carried out in our laboratory following a standardized methodology adapted from the official AOAC method (2006). The process involved kneading 50 g of semolina with 25 ml of distilled water for 15 min to obtain suitable dough for extrusion with moisture content of 30%. The dough was extruded using a laboratory press (Serma, Milan, Italy) to produce spaghetti with a thickness of 1.70 mm, which were then dried at 50 °C for 2 h. Pasta samples were cooked under controlled laboratory conditions using borosilicate glassware (Pyrex). All glassware used for cooking and handling samples was previously acid-cleaned by soaking in 10% (v/v) nitric acid and thoroughly rinsed with ultrapure water to avoid trace metal contamination. Finally, all samples (semolina and pasta) were oven-dried at 60 °C until constant weight and then milled for the determination of nutrients and phytic acid contents. All results are reported on a dry weight basis.

### Mineral Analysis

The nutrients analyzed in this study were Se, Zn, calcium (Ca), Fe, and Mg, selected based on their nutritional relevance and their known interaction with Se and Zn biofortification and phytic acid complexation. Sample preparation began with grinding using a ball mill (Retch PM 400) to achieve a particle size smaller than 300 μm. The ground samples then underwent acid digestion in a mixture of ultra-pure nitric acid and 30% hydrogen peroxide using a microwave digestion system (Mars X, CEM Corp, Matthews, NC, USA), as proposed by Zhao et al. (1994). Total nutrient concentrations in the digested samples were determined using inductively coupled plasma mass spectrometry (ICP-MS; Agilent Technologies, Santa Clara, USA) operating in hydrogen gas mode to minimize spectral interferences. Quality control was ensured through repeated analysis (*n* = 4) of a certified reference material (NIST 1573a sample, tomato leaf), digested and analyzed under identical conditions. Recovery rates for all analyzed elements exceeded 95%, indicating satisfactory analytical accuracy. Limits of detection (LLOD) and quantification (LOQ) were calculated from procedural blanks as three and ten times the standard deviation of the blank signal, respectively, and were well below the concentrations measured in the samples.

### Phytic Acid and Mineral Bioavailability

Phytic acid content was determined using a modified method from [39], measuring Fe in the supernatant after ferric phytate precipitation. Ground samples (0.2 g) were agitated in 10 mL of 0.2 M HCl (pH 3) for 2 h. Supernatant (1 mL) was mixed with 2 mL of NH_4_Fe(SO_4_)_2_·12H_2_O, heated for 30 min, cooled, and centrifuged at 3800 rpm for 3 min. Iron was measured by adding 1.5 mL of 0.064 M bipyridine to the supernatant, incubating for 10 min, and measuring absorbance at 419 nm. Potential mineral bioavailability was estimated by calculating molar ratios of phytic acid to Zn, Se, Ca, Fe and Mg [40].

Zinc bioavailability was further estimated through the total daily absorbed Zn (TAZ) and fractional absorption of Zn (FAZ) using the equations proposed by Miller et al. [41]:

For TAZ:

$$\begin{aligned}&TAZ=0.5*\Big{[}{A}_{MAX}+TDZ+{K}_{R}*\left(1+\frac{{R}_{PZ}*TDZ}{{K}_{p}}\right)\\&-\sqrt{{({A}_{MAX}+TDZ+{K}_{R}*(1+\frac{TDP}{{K}_{p}}\left)\right)}^{2}-4*{A}_{MAX}*TDZ}\Big{]}\end{aligned}$$

For FAZ:

$$\begin{aligned}&FAZ=\left(\frac{0.5}{\mathrm{TDZ}}\right){\cdot} \Big{[}{\mathrm{A}}_{\mathrm{MAX}}+\mathrm{TDZ}+{\mathrm{K}}_{\mathrm{R}}{\cdot} \left(1+\frac{\mathrm{TDP}}{{\mathrm{K}}_{\mathrm{P}}}\right)\\&-\sqrt{{\left({\mathrm{A}}_{\mathrm{MAX}}+\mathrm{TDZ}+{\mathrm{K}}_{\mathrm{R}}{\cdot} \left(1+\frac{\mathrm{TDP}}{{\mathrm{K}}_{\mathrm{P}}}\right)\right)}^{2}-4 {\cdot} {\mathrm{A}}_{\mathrm{MAX}}{\cdot} \mathrm{TDZ}}\Big{]}\end{aligned}$$

Where A_MAX_ = Maximum absorption; TDZ = Total Daily Dietary Zn (mmol d^− 1^); TDP = Total Daily Dietary Phytate (considering a consumption of pasta of 70 g dry pasta d^− 1^); K_R_ = Equilibrium dissociation constant of Zn-receptor binding; K_P_ = Equilibrium dissociation constant of Zn-phytate binding.

### Statistical Analysis

The effects of year, soil Zn application, foliar application, and their interactions on each parameter evaluated in semolina and pasta were analyzed jointly across years using mixed models with a split–split–plot ANOVA design. Year was considered the main-plot factor, soil Zn application the subplot factor, and foliar application the sub-subplot factor. Replication was included as a random effect. Although soil Zn was applied only at the beginning of the first growing season, it was retained as a treatment factor in the model to account for residual and carryover effects across years. The inclusion of year and its interactions with soil and foliar factors allowed explicit testing of interannual variability and differential treatment responses associated with climatic conditions and nutrient redistribution. Therefore, no independent year-by-year analyses were conducted. When significant effects were detected, mean comparisons were performed using Tukey’s honestly significant difference (HSD) test at *p* ≤ 0.05. Homogeneity of variances and normality of residuals were assessed using Levene’s test and the Shapiro–Wilk test, respectively. Linear regression analyses were used to examine the relationships between Zn and Se concentrations in grain and their corresponding levels in semolina and pasta. All statistical analyses were performed using Statistix v. 8.10 (Analytical Software, Tallahassee, FL, USA).

## Results

### Semolina Yield

Semolina yield was significantly influenced by both the year of study and the foliar application of nutrients (Se, Zn, or their combination), as indicated by the ANOVA results in Table 2. In contrast, soil Zn application and all interaction terms had no significant effects on semolina yield.

**Table 2:** Summary of split-split-plot analysis of variance (ANOVA) showing the effects of year, soil Zn application, foliar application, and their interactions on semolina (*n* = 4) and pasta (*n* = 2) parameters

		Year (Y)	Zn Soil (S)	Foliar (F)	Y×S	Y×F	S×F	Y×S×F
	df	1	1	1	3	3	3	3
Yield	Semolina	648.80***	0.01	4.49**	3.76	0.05	1.62	0.43
Zn	Semolina	19.38***	0.22	148.08***	20.30***	0.35	0.48	3.82
	Pasta	4.21	1.72	116.28***	0.61	0.16	0.37	0.11
Se	Semolina	7.92*	0.09	130.95***	22.19***	0.31	0.21	1.44
	Pasta	1.12	6.01*	239.28***	0.17	0.19	4.29*	0.71
Ca	Semolina	2.33	0.1	0.51	0.1	0.67	0.95	1.58
	Pasta	0.11	11.37**	12.67***	0.31	1.08	3.65*	0.48
Fe	Semolina	169.20***	0.91	0.62	1.27	1.9	0.38	0.86
	Pasta	12.51*	1.48	6.80**	1.46	0.36	4.56*	0.34
Mg	Semolina	254.39***	0.01	2.21	2.03	1.48	0.82	3.89*
	Pasta	3.64	9.46**	11.47***	0.16	0.07	0.76	0.37
PA	Semolina	12.66**	0.56	1.72	0.02	1.21	0.66	0.08
	Pasta	39.51***	0.49	5.00*	0.21	0.95	0.28	1.15
PA: Zn	Semolina	8.66**	0.27	98.74***	8.14**	0.81	0.5	0.70
	Pasta	0.17	0.82	58.09***	0.7	0.23	0.35	0.34
PA: Se	Semolina	8.5	0.4	46.39***	7.94**	1.6	0.49	0.18
	Pasta	8.15*	0.44	139.92***	0.05	0.97	3.91*	1.69
PA: Ca	Semolina	0.01	0.01	0.52	0.13	0.4	0.95	1.4
	Pasta	15.11**	20.02***	28.63***	1.12	0.41	4.47*	0.44
PA: Fe	Semolina	125.19***	0.89	0.63	1.52	1.42	0.67	1.08
	Pasta	5.58*	1.24	14.55***	1.81	0.43	6.73**	0.42
PA: Mg	Semolina	161.04***	0.12	2.15	1.58	0.56	0.79	2.51
	Pasta	0.23	10.89**	13.70***	0.05	0.1	0.62	0.52
FAZ	Semolina	35.94***	0.03	165.06***	21.68***	0.47	0.85	2.94*
	Pasta	7.11*	1.36	119.36***	1.37	0.12	0.26	0.22
TAZ	Semolina	16.14***	0.41	136.44***	17.74***	0.36	0.44	3.64*
	Pasta	2.26	1.999	97.80***	0.37	0.15	0.45	0.15

For each parameter, F-values are presented with significance levels (* *p* ≤ 0.05, ** *p* ≤ 0.01, *** *p* ≤ 0.001). df = degrees of freedom.

**Fig. 1:**
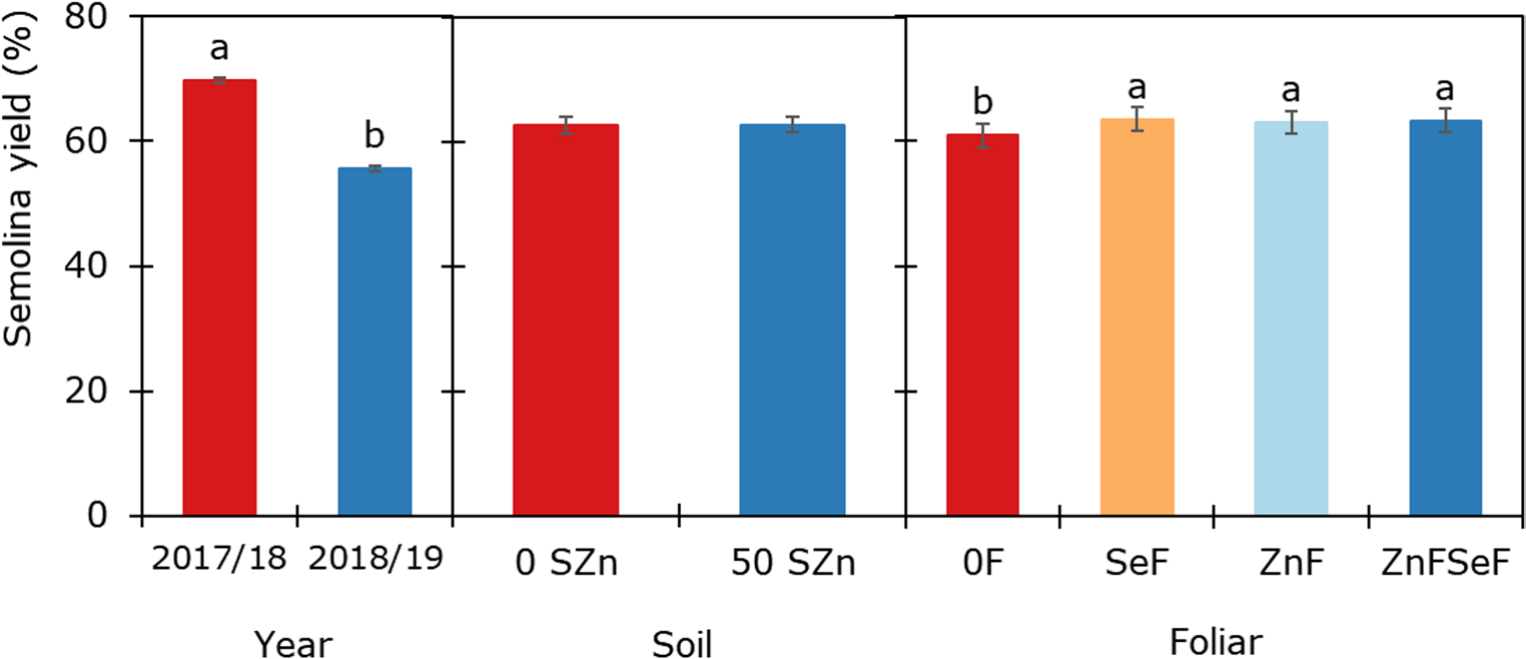
Effects of soil (S) and foliar (F) applications on semolina yield. Bars represent means (*n* = 4) with error bars indicating standard error. Different letters denote significant differences between treatments according to Tukey’s honestly significant difference (HSD) test (*p* ≤ 0.05)

Year had a strong effect on semolina yield, with significantly higher values recorded in the first growing season (2017/18) compared to the second (2018/19). As shown in Fig. 1, semolina yield in 2017/18 was 25.5% higher than in 2018/19, reflecting the contrasting environmental conditions between seasons. Soil application of Zn did not result in any observable effect, as yields were nearly identical to the control (approximately 63% in both cases). In contrast, foliar application, whether with Se, Zn, or their combination, consistently led to a significant increase in semolina yield, ranging from 3.37% to 4.15% over the control. No significant differences were detected among the individual foliar treatments.

### Zinc and Se Concentrations and Proxy Indices of Bioavailability

The analysis of variance shown in Table 2 revealed that Se and Zn concentrations in semolina were significantly affected by the year and, most notably, by foliar application, with a significant interaction between year and soil Zn application also observed. In pasta, Zn concentration was significantly influenced by foliar application, whereas Se was affected by soil Zn and foliar applications, as well as their interaction. Phytic acid (PA) content in semolina was significantly affected by the year of study, but not by soil Zn or foliar application. In pasta, both year and foliar application had significant effects on PA content.

For the PA: Zn molar ratio, significant effects were observed in semolina for year, foliar application, and the year × soil Zn interaction; while in pasta, only foliar application was significant. In the case of PA: Se ratio, semolina was significantly influenced by foliar application and the year × soil Zn interaction, whereas in pasta, year, foliar application, and the soil Zn × foliar interaction were all significant. Regarding the modeled indices of Zn absorption, both FAZ and TAZ in semolina were significantly affected by year, foliar application, year × soil and the triple interaction. In pasta, only year and foliar application had significant effects on FAZ, while TAZ was only significantly influenced by foliar application.

As can be seen in Table 3., Zn concentration in semolina was 11.5% higher in 2018/19 (30.66 mg kg⁻¹) than in 2017/18 (27.49 mg kg⁻¹), whereas Se concentration was approximately 6.8% higher in 2017/18. Foliar treatments markedly increased Zn concentration in semolina, with the combined foliar ZnFSeF treatment resulting in the highest values (36.72 mg kg⁻¹), representing a 75% increase compared to the control (20.99 mg kg⁻¹). Similarly, ZnF alone increased Zn concentration (34.83 mg kg⁻¹) by approximately 66% relative to the control. A comparable trend was observed for Se concentration in semolina, which peaked under ZnFSeF (43.10 µg kg⁻¹) and SeF (40.90 µg kg⁻¹), corresponding to increases of 75% and 66%, respectively, compared to the control (24.61 µg kg⁻¹). In pasta, Zn content was primarily affected by foliar Zn supply, with ZnFSeF (32.73 mg kg⁻¹) and ZnF (30.4 mg kg⁻¹) increasing Zn levels by 55% and 44%, respectively, relative to the control (21.10 mg kg⁻¹). Selenium content in pasta followed a similar trend, with ZnFSeF (41.56 µg kg⁻¹) and SeF (37.16 µg kg⁻¹) showing the highest values, corresponding to increases exceeding 160%, compared to the control (15.68 µg kg⁻¹).

**Table 3.** Effects of “year (Y)” and “foliar treatments (F)” on phytic acid (PA), Zn and Se concentrations and their bioavailability indices in semolina and pasta, including the PA:nutrient ratios, the total daily absorbed Zn (TAZ, expressed as per-unit) and fractional absorption of Zn (FAZ). Values are expressed as mean ± standard error

		Year		Foliar			
		2017/18	2018/19	No	SeF	ZnF	ZnFSeF
**Zn** (mg kg^− 1^)	Semolina	27.5 ± 1.4 b	30.7 ± 1.3 a	21.0 ± 0.8 b	23.7 ± 0.9 b	34.8 ± 0.8 a	36.7 ± 1.0 a
	Pasta	25.5 ± 1.5	26.9 ± 1.5	21.1 ± 0.6 c	20.3 ± 0.6 c	30.7 ± 0.6 b	32.7 ± 0.5 a
**Se** (µg kg^− 1^)	Semolina	35.3 ± 1.5 a	33.0 ± 1.7 b	24.6 ± 0.9 c	40.9 ± 0.9 a	27.9 ± 1.0 b	43.1 ± 1.0 a
	Pasta	28.2 ± 3.1	27.2 ± 3.1	15.7 ± 0.8 c	37.2 ± 1.3 b	16.4 ± 0.9 c	41.6 ± 1.2 a
**PA** **(**mg kg^− 1^)	Semolina	8.15 ± 1.90 b	8.32 ± 1.54 a	8.26 ± 2.07	8.28 ± 1.93	8.14 ± 0.52	8.26 ± 0.72
	Pasta	7.87 ± 0.00 b	8.45 ± 0.01 a	8.32 ± 0.00 ab	8.36 ± 0.00 a	8.00 ± 0.00 ab	7.97 ± 0.00 b
**PA: Zn**	Semolina	31.94 ± 1.90	29.38 ± 1.54	39.76 ± 2.07 a	35.01 ± 1.93 b	22.79 ± 0.52 c	25.07 ± 0.72 c
	Pasta	32.28 ± 2.19	32.56 ± 1.99	39.13 ± 1.32 a	40.80 ± 1.54 a	25.76 ± 0.47 b	23.99 ± 0.40 b
**PA: Se**	Semolina	0.029 ± 0.001	0.033 ± 0.002	0.041 ± 0.002 a	0.024 ± 0.001 c	0.036 ± 0.001 b	0.023 ± 0.001 c
	Pasta	0.041 ± 0.005 b	0.046 ± 0.005 a	0.065 ± 0.003 a	0.027 ± 0.001 b	0.059 ± 0.003 a	0.023 ± 0.001 b
**FAZ** (g day^− 1^)	Semolina	26.64 ± 0.13 a	26.18 ± 0.16 b	27.40 ± 0.06 a	27.14 ± 0.09 a	25.67 ± 0.01 b	25.43 ± 0.07 b
	Pasta	32.08 ± 0.75 a	31.28 ± 0.76 b	34.50 ± 0.32 a	34.51 ± 0.31 a	29.25 ± 0.30 b	28.46 ± 0.22 b
**TAZ**	Semolina	0.74 ± 0.00 a	0.80 ± 0.00 b	0.60 ± 0.01 b	0.64 ± 0.02 b	0.89 ± 0.00 a	0.93 ± 0.01 a
	Pasta	0.80 ± 0.03	0.82 ± 0.03	0.70 ± 0.01 b	0.70 ± 0.01 b	0.90 ± 0.01 a	0.93 ± 0.01 a

Within each parameter and factor, different letters mean significant differences between means according to the Tukey’s honestly significant difference (HSD) test (*p* ≤ 0.05).

**Fig. 2:**
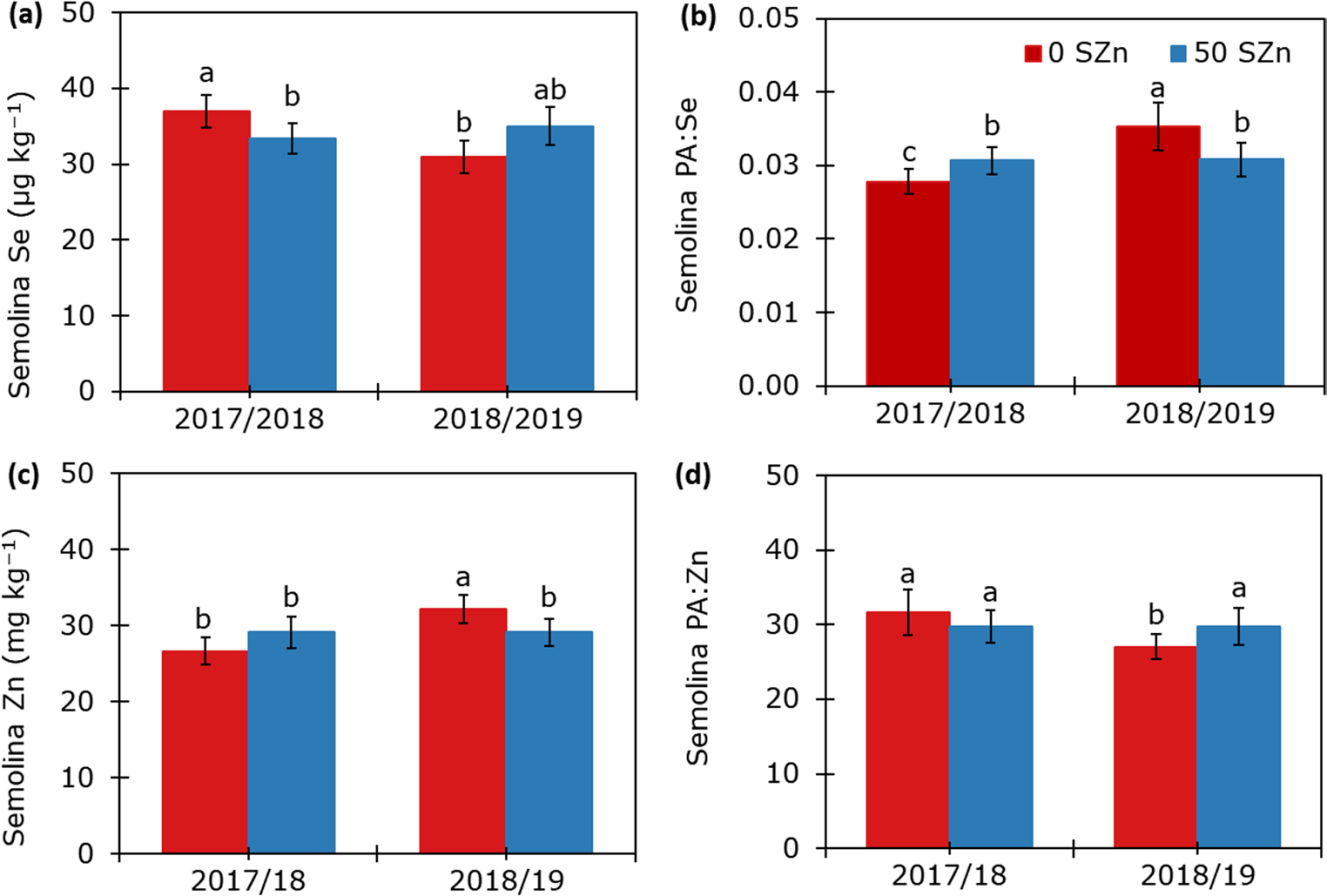
Effect of the interaction “year × soil” (Y × S) on (**a**) Se and (**b**) Zn concentration, (**c**) phytate: Se and (**d**) phytate: Zn ratios in semolina. Bars indicate means (*n* = 4), error bars with error bars indicating standard error. Different letters denote significant differences between treatments according to Tukey’s honestly significant difference (HSD) test (*p* ≤ 0.05)

Regarding the year × soil interaction in semolina, Se concentration (Fig. 2a), values were 10.7% higher without soil Zn in 2017/18, while in 2018/19 Se concentration increased by 13.1% following soil Zn application. On the other hand, Zn concentration increased by 9.4% with soil Zn application in 2017/18, whereas in 2018/19 the highest Zn levels were observed in the absence of soil Zn, with a 9.7% decrease following soil Zn application (Fig. 2c).

Phytic acid (PA) content in semolina was slightly (2.08%) but significantly higher in 2018/19 than in 2017/18, with no significant differences among foliar treatments. In pasta, PA content was also higher, about 7.34%, in 2018/19 compared to 2017/18. Notably, pasta produced from the Control and ZnF/ZnFSeF treatments had lower PA levels compared to SeF alone (Table 3.).

The PA: Se ratio was lowest in ZnFSeF and SeF treatments in both semolina and pasta, with reductions of up to 44%. Regarding the year × soil interaction (Fig. 2b), the PA: Se ratio showed being lowest in 2017/18 without soil Zn (0.028) and highest in 2018/19 without soil Zn (0.035), with soil Zn application reducing the ratio by approximately 11% in the latter year (0.031). The PA: Zn molar ratio was significantly reduced by foliar Zn application. In semolina, ZnF (22.79) and ZnFSeF (25.07) reduced the PA: Zn ratio by 43% and 37%, respectively, compared with the control (39.76) (Table 3.). In pasta, reductions of 34% and 39% were observed under the same treatments. The application of Zn to the soil significantly increased the PA: Zn ratio in 2018/19 but not in 2017/18 (Fig. 2d).

Fractional absorption of Zn (FAZ) in both semolina and pasta was highest in the control and SeF treatments and lowest under ZnF and ZnFSeF. In contrast, total absorbed Zn (TAZ) was highest under ZnFSeF and ZnF treatments. In semolina, TAZ increased by 55% in ZnFSeF and 48% in ZnF relative to the control, while in pasta increases of 33% were observed in both treatments (Table 3.).

**Fig. 3:**
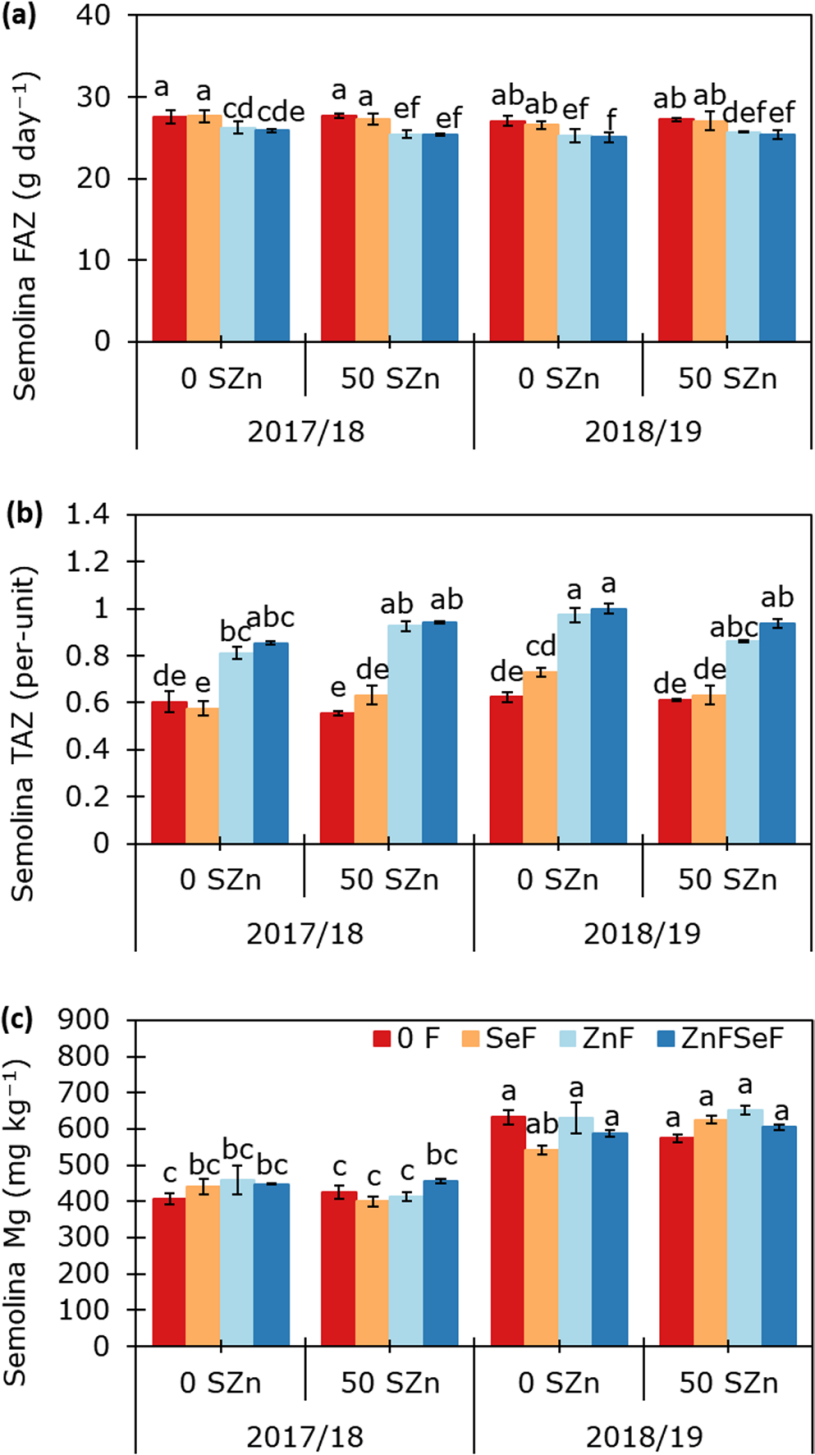
Effect of the interaction “year × soil × foliar” (Y × S × F) on (**a**) Mg concentration and (**b**) fractional absorption of Zn (FAZ) in semolina and (**c**) total daily absorbed Zn (TAZ) in pasta. Bars represent means (*n* = 4) with error bars indicating standard error. Different letters denote significant differences between treatments according to Tukey’s honestly significant difference (HSD) test (*p* ≤ 0.05)

In semolina, FAZ was generally higher in the control and SeF treatments and lower in ZnF and ZnFSeF, with minor differences due to soil Zn or year (Fig. 3a). Total absorbed Zn (TAZ) in semolina was highest in ZnFSeF and ZnF treatments, particularly in 2018/19, and was further enhanced by soil Zn application (Fig. 3b).

### Ca, Fe and Mg Concentration and Proxy Indices of Bioavailability

Table 2 shows the effect of the main factors and their interactions on the Ca, Fe and Mg concentrations and their associated proxy indices of bioavailability in semolina and pasta. For Ca, no significant effects were observed in semolina, whereas in pasta, soil Zn, foliar application and their interaction significantly affected Ca concentration. Iron concentration in semolina was significantly affected by year, while in pasta, both year and foliar application were significant, together with the soil Zn × foliar interaction. For Mg, semolina concentrations were significantly affected by the year of study and the triple interaction, while in pasta, both soil Zn and foliar application were significant.

The PA: Ca ratio showed no significant effects in semolina, but in pasta, year, soil Zn, and foliar application were all significant, together with the soil Zn × foliar interaction. The PA: Fe ratio in semolina was significantly affected by year, while in pasta, year, foliar application, and the soil Zn × foliar interaction were significant. For the PA: Mg ratio, semolina was also significantly influenced by year, while in pasta, both soil Zn and foliar application were significant (Table 2).

As shown in Table 4, Ca concentration in pasta increased significantly in response to foliar Zn treatments. The highest Ca concentrations were observed under ZnFSeF and ZnF treatments (with an average content of 188.5 mg kg⁻¹), representing an increase of approximately 12% compared to the control and SeF treatments (168.5 mg kg⁻¹).

**Table 4:** Effect of “year (Y)” and “foliar treatments (F)” on Ca, Mg and Fe concentrations and their bioavailability indices in semolina and pasta. Values are expressed as mean ± standard error

		Year		Foliar			
		2018	2019	No	SeF	ZnF	ZnFSeF
**Ca** (mg kg^− 1^)	Semolina	218.0 ± 2.1	224.3 ± 3.5	225.2 ± 4.3	220.2 ± 3.8	221.0 ± 5.1	218.3 ± 3.0
	Pasta	177.9 ± 3.6	179.0 ± 4.0	167.5 ± 5.9 b	169.5 ± 4.0 b	186.5 ± 3.7 a	190.4 ± 1.7 a
**Fe** (mg kg^− 1^)	Semolina	15.1 ± 0.3 b	19.9 ± 0.3 a	17.1 ± 0.7	17.4 ± 0.7	17.6 ± 0.9	17.8 ± 0.6
	Pasta	13.9 ± 0.6 b	16.4 ± 0.7 a	16.5 ± 0.8	12.6 ± 0.9	16.3 ± 1.1	15.2 ± 0.7
**Mg** (mg kg^− 1^)	Semolina	431.3 ± 1.5 b	606.1 ± 1.7 a	510.0 ± 0.9	501.8 ± 0.9	538.8 ± 1.0	524.3 ± 1.0
	Pasta	324.5 ± 9.9	342.9 ± 10.0	303.4 ± 8.9 c	312.2 ± 11.3 c	343.9 ± 14.5 b	375.3 ± 4.8 a
**PA: Ca**	Semolina	2.27 ± 0.02	2.31 ± 0.03	2.26 ± 0.04	2.29 ± 0.04	2.30 ± 0.04	2.31 ± 0.02
	Pasta	2.70 ± 0.07	2.89 ± 0.08	3.03 ± 0.11 a	3.00 ± 0.09 a	2.60 ± 0.05 b	2.54 ± 0.04 b
**PA: Fe**	Semolina	4.61 ± 0.08 a	3.75 ± 0.09 b	4.24 ± 0.13	4.18 ± 0.19	4.12 ± 0.15	4.18 ± 0.13
	Pasta	4.94 ± 0.22 a	4.51 ± 0.21 b	4.34 ± 0.19 b	5.76 ± 0.27 a	4.30 ± 0.30 b	4.51 ± 0.16 b
**PA: Mg**	Semolina	0.69 ± 0.01 a	0.51 ± 0.02 b	0.63 ± 0.03	0.64 ± 0.03	0.59 ± 0.03	0.55 ± 0.04
	Pasta	0.90 ± 0.03	0.91 ± 0.03	1.00 ± 0.03 a	0.98 ± 0.04 ab	0.86 ± 0.04 bc	0.77 ± 0.01 c

Within each parameter and factor, different letters mean significant differences between means according to the Tukey’s honestly significant difference (HSD) test (*p* ≤ 0.05).

**Fig. 4:**
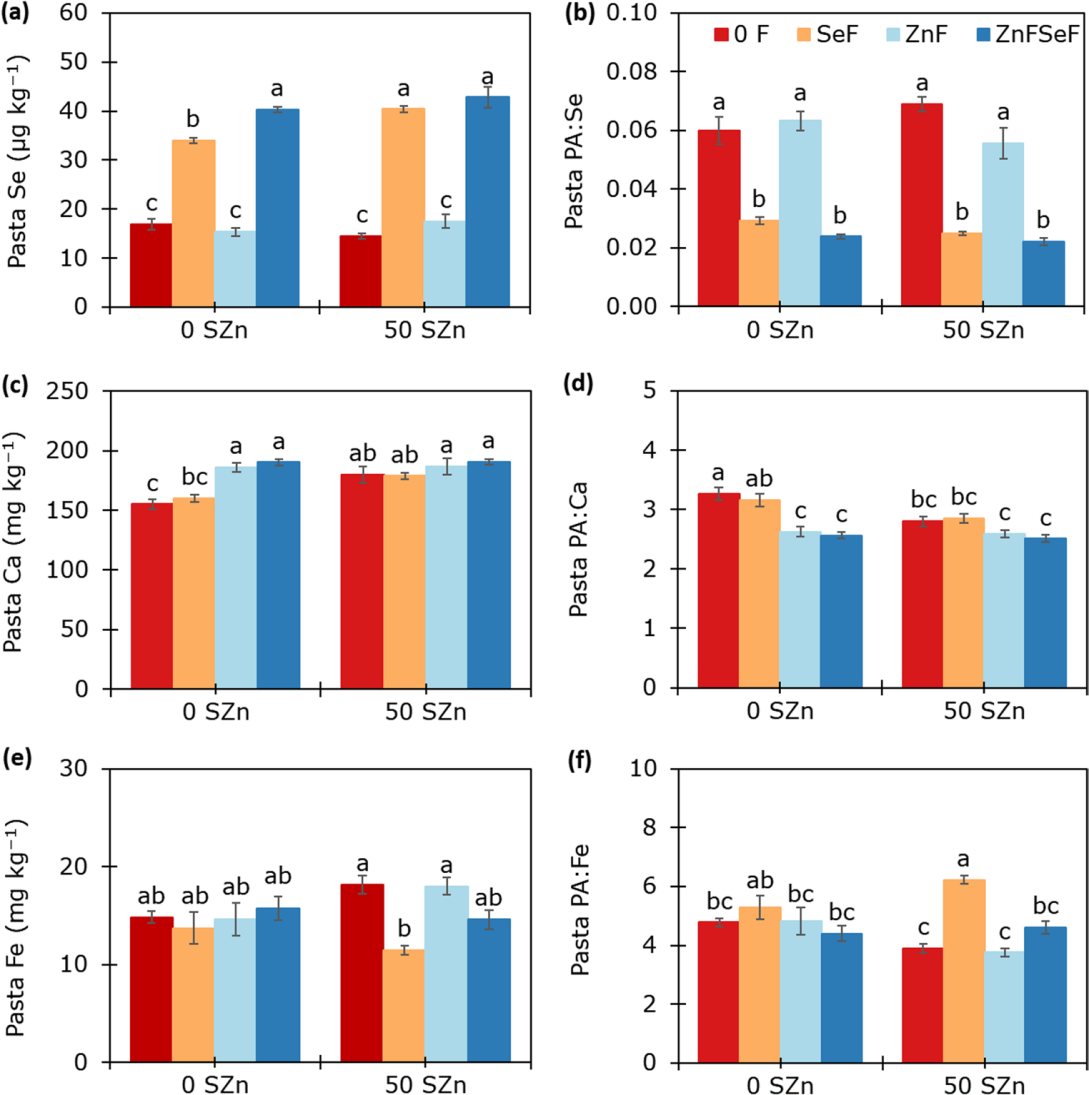
Effects of the interaction “soil × foliar” (S × F) on (**a**) Se, (**b**) Ca and (**c**) Fe concentrations, and (**d**) phytate: Se, (**e**) phytate: Ca and (**f**) phytate: Fe ratios in pasta. Bars represent means (*n* = 4) with error bars indicating standard error. Different letters denote significant differences between treatments according to Tukey’s honestly significant difference (HSD) test (*p* ≤ 0.05)

The soil × foliar interaction further indicated that Ca concentration was lowest in the control and SeF treatments under non-amended soil conditions (Fig. 4b). In contrast, Ca concentration in semolina remained stable across years and treatments, with mean values close to 221 mg kg⁻¹. Iron concentration showed a pronounced year effect. In both semolina and pasta, Fe concentration was 31.8% and 18.0% higher, respectively, in 2018/19 than in 2017/18. The soil Zn × foliar interaction revealed higher Fe concentrations in semolina under the combined application of soil Zn with either no foliar treatment or ZnF (Fig. 4c). Finally, Mg concentration in semolina was 40,5% higher in 2018/19 compared to 2017/18. In pasta, both soil Zn and foliar treatments significantly affected Mg content, with the highest concentration observed in the combined Zn and Se foliar treatment (ZnFSeF: 375.3 mg kg⁻¹), corresponding to an increase of 23.7% compared to the control, followed by ZnF (343.9 mg kg⁻¹, 13.3% increase).

The PA: Ca ratio in semolina was not significantly affected by any factor. In pasta, however, ZnF and ZnFSeF treatments reduced the PA: Ca ratio by 14.2% and 16.2%, respectively, compared to the control, and by 13.3% and 15.3% relative to SeF, with or without soil Zn application (Table 4; Fig. 4e). The PA: Fe ratio was significantly lower in 2018/19 than in 2017/18 in semolina and pasta, with reductions of 18.7% in semolina and 8.7% in pasta, respectively. The PA: Mg ratio in semolina was significantly lower in 2018/19 than in 2017/18, concretely 26.1%, whereas in pasta, both ZnF and ZnFSeF significantly reduced the ratio (0.86 and 0.77, respectively) compared to the control (1.00) and SeF (0.98).

###  Grain-to-Product Transfer Efficiency

The relationship between Zn and Se concentrations in grain and their corresponding levels in semolina and pasta revealed a strong and consistent grain-to-product transfer pattern for both micronutrients. For semolina, linear regression analyses showed that grain Zn concentration explained 74% of the variability in semolina Zn content (R² = 0.744), with an average semolina/grain ratio of 0.74. Similarly, Se concentration in semolina exhibited an even stronger association with grain Se content (R² = 0.89), with a comparable semolina/grain ratio of 0.74, indicating a highly consistent transfer during the milling process.

**Fig. 5:**
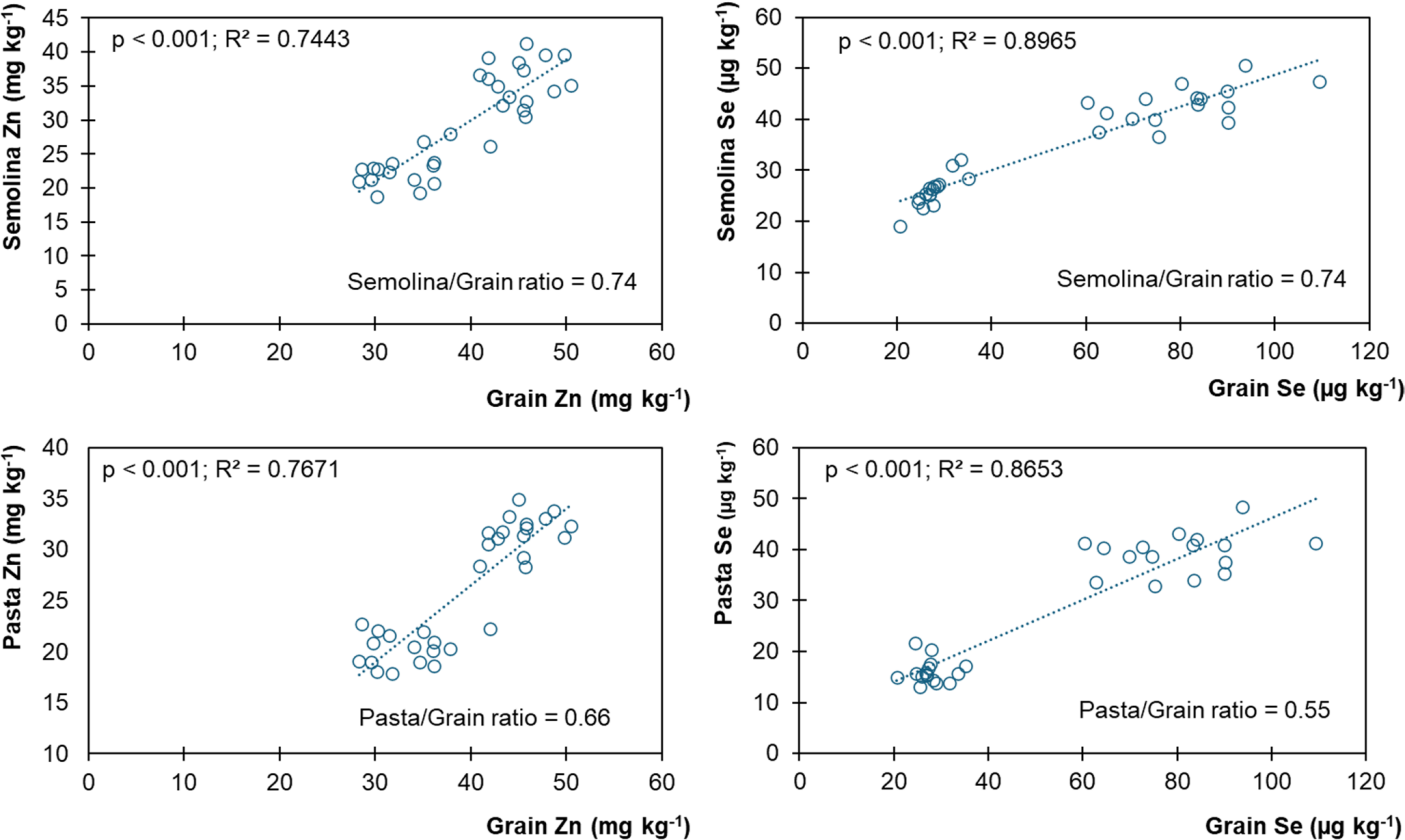
Relationships between Zn (left) or Se (right) concentrations in grain and their respective concentrations in semolina and pasta. Each point represents one experimental plot; semolina and pasta values are derived from the same grain sample. Lines indicate linear trends describing micronutrient retention during processing. Pearson’s correlation coefficient (r) and the coefficient of determination (R²) are shown for descriptive purposes

In pasta, grain Zn concentration accounted for 77% of the variability in pasta Zn content (R² = 0.767), with an average pasta/grain ratio of 0.66, suggesting that approximately two-thirds of grain Zn was conserved in the dry pasta product. For Se, the relationship between grain and pasta concentrations remained strong (R² = 0.865), although the pasta/grain ratio was lower (0.55), reflecting greater processing-related losses for Se compared to Zn during pasta production. (Figure 5.)

## Discussion

This study demonstrates that agronomic biofortification with Zn and Se is a viable and robust strategy for improving the nutritional profile of durum wheat semolina and pasta under Mediterranean dryland conditions when evaluated in terms of micronutrient enrichment and proxy indices of bioavailability. The positive effects observed were primarily associated with foliar applications, whereas soil Zn fertilization showed limited and context-dependent responses, reflecting the complexity of micronutrient behavior under rainfed Mediterranean systems. Moreover, the effectiveness of this biofortification program was strongly influenced by interannual climatic variability, highlighting the importance of environmental factors in determining micronutrient dynamics and grain quality.

### Effects of Biofortification and Climate Conditions on Semolina Yield

Semolina yield was significantly affected by both micronutrient applications and growing season conditions. Foliar treatments of Se (SeF), Zn (ZnF), and their combination (ZnFSeF) led to statistically significant increases of approximately 3.2 to 4.1% in semolina yield compared to the control, indicating a moderate but consistent improvement in milling extractability under the tested conditions.

These increases can be partially attributed to the physiological roles of Zn and Se in grain development. Zinc supports protein synthesis, starch formation, and enzyme activation, and therefore, must be transferred to endosperm to promote its development [42, 43]. Although the exact transfer mechanisms remain to be fully elucidated [44], Se application has been reported to boost antioxidant activity and stress tolerance during grain filling in wheat [45]. Under Mediterranean dryland conditions, these effects may contribute to a more stable grain filling process, potentially improving grain physical characteristics relevant to semolina extraction, even in the absence of direct measurements of industrial quality traits.

A strong seasonal effect was observed across the studied period: average semolina yield in the 2018/19 season was substantially lower than in 2017/18. This reduction coincided with lower rainfall (295 mm vs. 477 mm) and higher temperatures during critical developmental stages, suggesting that heat and drought stress negatively affected grain characteristics such as starch deposition, protein profile, and vitreousness, all of which influence extractability. These findings align with previous studies reporting semolina yield reductions of 15–25% under similar drought conditions in Mediterranean and semiarid environments [46, 47]. Despite this variability, the positive effects of foliar Zn and Se were consistent across seasons, as evidenced by the non-significant interaction between year and foliar treatment. This consistency may suggest that foliar biofortification could partially buffer the negative effects of adverse climatic conditions on semolina extraction efficiency, although it would not offset the key influence of water availability and temperatures on overall milling performance. This is particularly relevant in the context of increasing climate uncertainty.

### Impact of Biofortification on Se and Zn Concentrations and Bioavailability Indices

The enrichment patterns in semolina and pasta could be explained by the distinct uptake and transport pathways for Se and Zn in durum wheat. It has been previously reported that Se, applied as selenate, is efficiently taken up via sulfate transporters and demonstrates preferential transport to shoots, in contrast to selenite forms, which tend to remain in roots [48]. This could explain why foliar application of selenate (SeF) was particularly effective at increasing grain Se concentration, which subsequently translated to increased Se levels in semolina and pasta. In parallel, foliar Zn application (ZnF) could have bypassed soil-related limitations by delivering Zn directly to photosynthetically active tissues, in agreement with previous studies on biofortification [49–53]. While Zn typically accumulates in the aleurone layer and embryo and it is then partially removed during milling [32], our results indicate that important quantities were retained in semolina and pasta, leading to significant increases relative to the control.

The combined foliar treatment (ZnFSeF) yielded the highest concentrations of both micronutrients in the final products and these values were significantly higher than those of the individual applications by approximately 11% for Se and 7% for Zn. This additive response may reflect complementary physiological effects reported in the literature, such as Se-mediated improvement of antioxidant capacity through increased superoxide dismutase activity [54] and Zn-induced modulation of sulfate transporters that could facilitate Se uptake [55]. However, these mechanisms were not directly assessed and therefore should be confirmed in future studies.

From a practical agricultural perspective, the combined foliar application of Zn and Se at flowering emerges as a robust strategy to improve the nutritional quality of durum wheat-derived products under Mediterranean rainfed conditions, even in seasons characterized by contrasting rainfall patterns. While previous studies have shown that adequate Se and Zn supply enhances crop yield and nutritional quality [56, 57], our results extend this evidence by demonstrating that such improvements are effectively transferred to semolina and pasta, highlighting the relevance of this approach beyond the field and into the food chain. Nevertheless, as standard industrial processing quality traits were not evaluated, these benefits should be interpreted in nutritional rather than technological terms.

### Phytic Acid (PA) Dynamics and Mineral Interactions

Beyond total micronutrient concentration, the nutritional relevance of biofortification depends on mineral bioavailability, which in cereal-based foods is strongly influenced by phytic acid (PA) due to its strong chelating properties of divalent cations such as Zn²⁺, Ca²⁺, Mg²⁺, and Fe²⁺ [58]. Consequently, PA: mineral molar ratios serve as proxy indicators of potential mineral bioavailability, with lower values indicating improved nutritional quality [58].

In the present study, PA was quantified using a colorimetric precipitation method based on ferric phytate formation. While this approach is widely applied in cereal and pasta matrices, it may be affected by matrix effects and co-precipitation phenomena. For this reason, PA and derived molar ratios were interpreted as relative indicators of changes in potential mineral bioavailability. Although future studies incorporating chromatographic or enzymatic methods would allow a more detailed nutritional assessment, the applied methodology remains appropriate for evaluating relative treatment effects in agronomic biofortification studies.

Similarly, Se and Zn were applied as sodium selenate and zinc sulphate, respectively, and mineral enrichment was evaluated on the basis of total concentrations and PA-related indices. While chemical speciation was not assessed, this approach allows an integrated assessment of biofortification effectiveness at the food-product level, where milling and processing already imply major constraints on mineral retention and bioavailability. Moreover, previous studies have shown that agronomic application of selenate in cereals predominantly leads to the accumulation of organic Se species, such as selenomethionine, in the grain, which are considered nutritionally relevant forms for human consumption [59]. Thus, total Se concentration remains a useful indicator of biofortification efficiency at the agronomic scale.

Our results demonstrate that foliar Zn application, both alone (ZnF) and in combination with Se (ZnFSeF), significantly decreased PA: Zn molar ratios in semolina and pasta. Although these reductions did not fall below the critical threshold (PA: Zn < 15), they represent nutritionally meaningful improvements, as lower ratios are associated with substantially increased zinc absorption in humans [60]. This was further supported by modeled indices, total absorbed zinc (TAZ) and fractional absorption of zinc (FAZ), calculated using the Miller equation validated against human absorption studies [41]. The ZnFSeF treatment significantly improved these functional metrics, resulting in higher TAZ for the pasta. The observed divergence between FAZ and TAZ reflects well-established homeostatic regulation of Zn absorption, whereby fractional absorption declines as dietary Zn concentration increases, while total absorbed Zn may still rise due to higher intake. Consequently, despite the lower FAZ values recorded under ZnF and ZnFSeF treatments compared with the control, these treatments resulted in significantly higher TAZ, indicating a net improvement in Zn supply from the biofortified products. This response pattern is consistent with previous Zn biofortification studies and highlights TAZ as a more nutritionally meaningful indicator when comparing foods differing markedly in Zn concentration [61, 62].

From a dietary perspective, considering that the estimated average requirement (EAR) for Zn is 8.6–14.4 mg per day depending on dietary patterns [63], our biofortified pasta could potentially contribute 6 to 10% of daily Zn requirements from a typical 70 g serving, an important increase when compared to non-biofortified pasta. In contrast, PA: Se ratios were consistently low across all Se-containing treatments, indicating a high potential bioavailability of Se in the final products. This represents a relevant nutritional gain compared to non-biofortified pasta and supports the findings of several studies which demonstrated the importance of considering the bioavailability when modelling dietary patterns that meet nutrient requirements [64, 65].

### Effects on Other Minerals and Nutrient Interactions

While our biofortification strategy primarily focused on the improvement of the Zn and Se uptake, significant effects on other nutritionally relevant minerals were also observed, revealing complex nutrient interactions with important implications. Magnesium concentrations in pasta responded positively to foliar treatments, with significant increases observed in ZnF (13.4%) and ZnFSeF treatments (23.7%) compared to the control. Calcium also showed modest increases in pasta from ZnFSeF treatments, though the effect was less pronounced than for Mg and was not observed consistently across environments. Interestingly, the effect was significant in pasta but not in semolina, suggesting potential interactions during processing that might have improved Ca retention or bioavailability. This outcome consistent with the findings of previous literature, where foliar application of Zn increased the nutrient concentration of other nutrients like Mg in wheat [66]. This could be explained considering the effect of Zn in the development of the plant root system which could have facilitated greater nutrient acquisition. Additionally, it could have improved the photosynthetic efficiency of the wheat plants, leading to increased carbon allocation and the subsequent translocation of nutrients such as Mg or Ca [67]. In contrast to these positive responses, Fe concentrations showed no significant changes in either semolina or pasta across all biofortification treatments. This lack of response could be attributed to the fact that Fe is mainly accumulated in the aleurone layer [68], and the potential positive effect may have been reduced by milling process. This outcome confirms a known limitation of Zn- and Se-centered biofortification strategies and underscores the challenge of simultaneously addressing multiple micronutrient deficiencies within cereal-based systems.

### Grain-To-Product Transfer Efficiency: from Biofortified Grain to Consumer Pasta

The efficacy of biofortification strategies depends not only on increasing grain nutrient concentrations but also on maintaining these levels throughout milling and food processing. In this study, Se and Zn retention during was assessed during the transformation of biofortified durum wheat grain [69] to semolina and subsequently to pasta, revealing distinct element-specific patterns with important nutritional implications. The presented data describes relative transfer efficiencies of Se and Zn from grain to semolina and to dry pasta, rather than a complete retention balance across all processing steps.

Although milling resulted in important losses, biofortified semolina maintained significantly elevated nutrient levels compared to control samples, demonstrating that foliar application successfully increased micronutrient accumulation not only in the outer grain layers but also substantially in the endosperm, the primary constituent of semolina. Based on typical Mediterranean consumption patterns of 70–100 g pasta per day, the biofortified pasta from ZnFSeF treatments could provide approximately 2.3 to 3.3 mg of Zn and 2.9 to 4.2 µg of Se per serving. These amounts represent around 25% of the recommended daily allowance (RDA) for Zn (8 to 11 mg per day) [70] and 5.2 to 7.6% of the RDA for Se (55 µg per day) for adults [71], making substantial contributions toward addressing deficiencies that are prevalent throughout Mediterranean populations reliant on cereal-based diets.

## Conclusions

This study demonstrated that combined foliar application of Zn and Se is an effective and practical strategy for improving the nutritional quality of durum wheat semolina and pasta under Mediterranean rainfed conditions. The dual application of Zn and Se not only increased the concentrations of both micronutrients, but also improved estimated bioavailability, as evidenced by reduced phytic acid-to-mineral molar ratios and increased values of total absorbed Zn (TAZ). Among all treatments, the combined ZnFSeF foliar spray yielded the highest Zn and Se concentrations in pasta, outperforming individual applications and control treatments. Importantly, the nutritional improvements achieved through biofortification were maintained after milling and pasta processing, indicating effective transfer of both micronutrients into the edible end products without compromising semolina yield. From a dietary perspective, the enriched pasta could provide a meaningful contribution to daily Zn and Se requirements, which is particularly relevant in Mediterranean populations where cereal-based foods constitute a major component of the diet.

Overall, these findings support the integration of combined foliar Zn and Se biofortification into sustainable wheat production systems as a practical approach to addressing micronutrient deficiencies. Future research should further investigate micronutrient speciation, post-cooking retention, and consumer-level bioavailability to refine nutritional impact assessments and optimize biofortification strategies across diverse agro-environmental conditions.

## Supplementary Information

Below is the link to the electronic supplementary material.

Supplementary Material 1 (DOCX 2.59 MB) 

## Data Availability

The data supporting the findings of this study are available from the corresponding author if reasonable requests are made.
