# Supplementary material for: Combined Biofortification of Durum Wheat with Zinc and Selenium: Effects on Semolina and Pasta Nutrient Accumulation and Yield
Source: Biol Trace Elem Res. 2026 Mar 24;204(7):5670–84. doi: 10.1007/s12011-026-05060-2 (PMC13319702; doi:10.1007/s12011-026-05060-2)
Supplement: Supplementary file 1 — Supplementary Material 1 (DOCX 2.59 MB) [file 12011_2026_5060_MOESM1_ESM.docx]

**Journal: Biological Trace Element Research**

**Title: Combined Biofortification of Durum Wheat with Zinc and Selenium: Effects on Semolina and Pasta Nutrient Accumulation and Yield**

### **Carlos García-Latorre*, Angélica Rivera-Martín, María Dolores Reynolds-Marzal, Maria J. Poblaciones^1^**

Department of Agronomy and Forest Environment Engineering, University of Extremadura, 06006 Badajoz, Spain

[*cgarcialn@unex.es](mailto:*cgarcialn@unex.es), 660145292, <https://orcid.org/0000-0003-1256-6186>

**^1^**<https://orcid.org/0000-0002-1559-2382>

**Supplementary material**


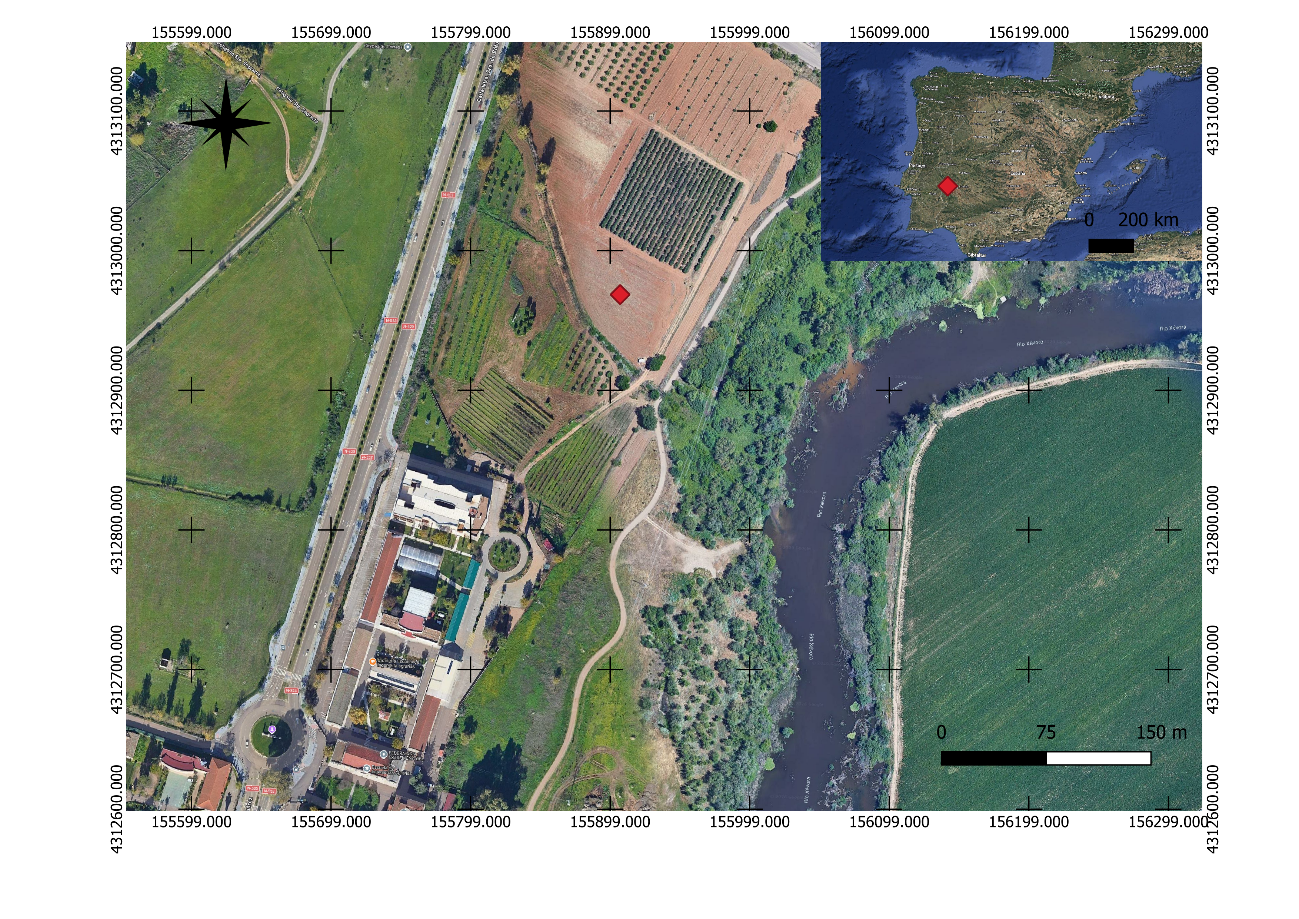


**Figure S1.** Study site location. The main map shows a high-resolution satellite view of the experimental plots (red diamond) with coordinates in WGS 84 (UTM zone 30N). The inset map displays the study site location within the Iberian Peninsula. The scale bars represent 150 m for the local site and 200 km for the regional context.


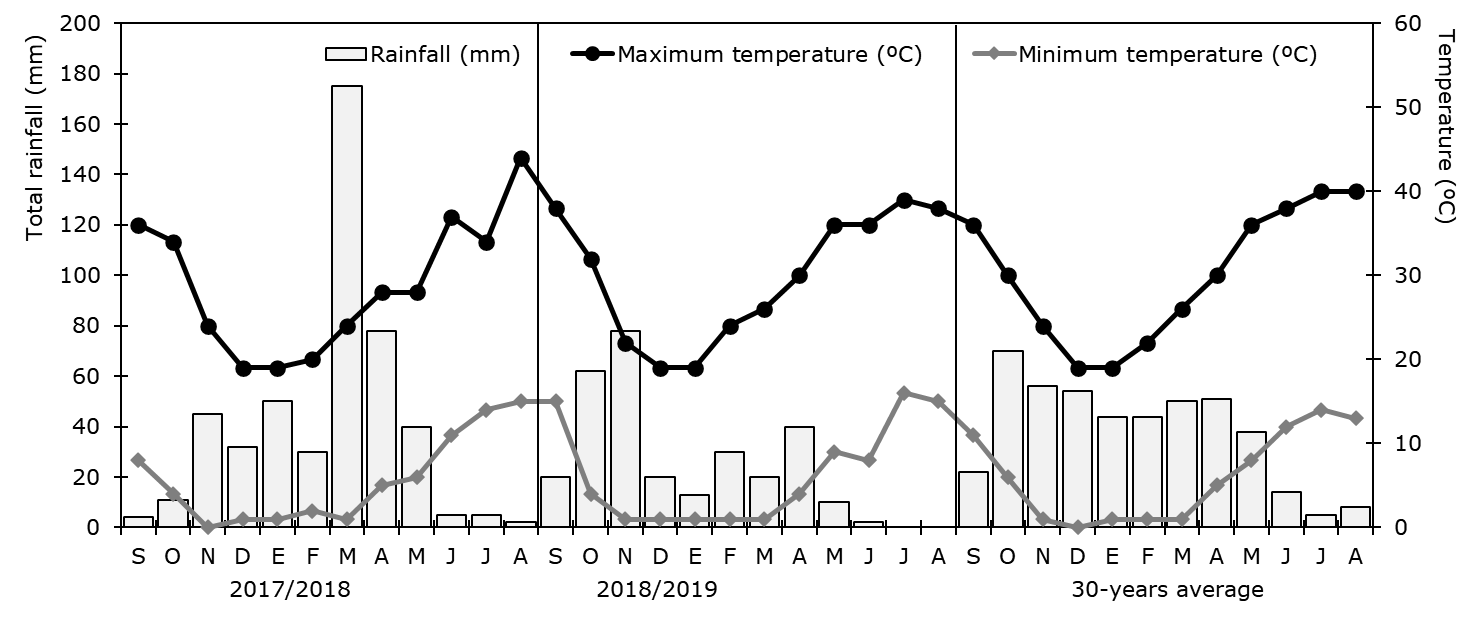


**Fig. S2.** Monthly meteorological conditions during the 2017/2018 and 2018/2019 growing seasons compared to the 30-year historical average at the study site. Bars represent total monthly rainfall (mm); lines with circles denote mean maximum temperature, and lines with diamonds denote mean minimum temperature (°C).
